# Overview on biomarkers for immune oncology drugs

**DOI:** 10.37349/etat.2025.1002298

**Published:** 2025-03-17

**Authors:** Evgeny N. Imyanitov, Elena V. Preobrazhenskaya, Natalia V. Mitiushkina

**Affiliations:** University of Salford, UK; ^1^Department of Tumor Growth Biology, N.N. Petrov Institute of Oncology, 197758 St.-Petersburg, Russia; ^2^Department of Medical Genetics, St.-Petersburg State Pediatric Medical University, 194100 St.-Petersburg, Russia

**Keywords:** Immune checkpoint inhibitors, cancer therapy, PD-L1/PD-1, microsatellite instability, tumor mutation burden, microbiome, neutrophil-to-lymphocyte ratio

## Abstract

Although immune checkpoint inhibitors (ICIs) are widely used in clinical oncology, less than half of treated cancer patients derive benefit from this therapy. Both tumor- and host-related variables are implicated in response to ICIs. The predictive value of PD-L1 expression is confined only to several cancer types, so this molecule is not an agnostic biomarker. Highly elevated tumor mutation burden (TMB) caused either by excessive carcinogenic exposure or by a deficiency in DNA repair is a reliable indicator for ICI efficacy, as exemplified by tumors with high-level microsatellite instability (MSI-H). Other potentially relevant tumor-related characteristics include gene expression signatures, pattern of tumor infiltration by immune cells, and, perhaps, some immune-response modifying somatic mutations. Host-related factors have not yet been comprehensively considered in relevant clinical trials. Microbiome composition, markers of systemic inflammation [e.g., neutrophil-to-lymphocyte ratio (NLR)], and human leucocyte antigen (HLA) diversity may influence the efficacy of ICIs. Studies on ICI biomarkers are likely to reveal modifiable tumor or host characteristics, which can be utilized to direct the antitumor immune defense. Examples of the latter approach include tumor priming to immune therapy by cytotoxic drugs and elevation of ICI efficacy by microbiome modification.

## Introduction

Immune checkpoint inhibitors (ICIs) were developed in the first decade of this century and rapidly became a standard for systemic therapy of many cancer types. The concept of ICIs is based on the assumption that malignant cells are generally recognizable by immunity, and, therefore, need to produce local immune suppressors in order to escape from host defense mechanisms. Consequently, therapeutic inactivation of these suppressors should restore anticancer immunity and eventually eliminate transformed cells [1, 2].

**Figure 1:**
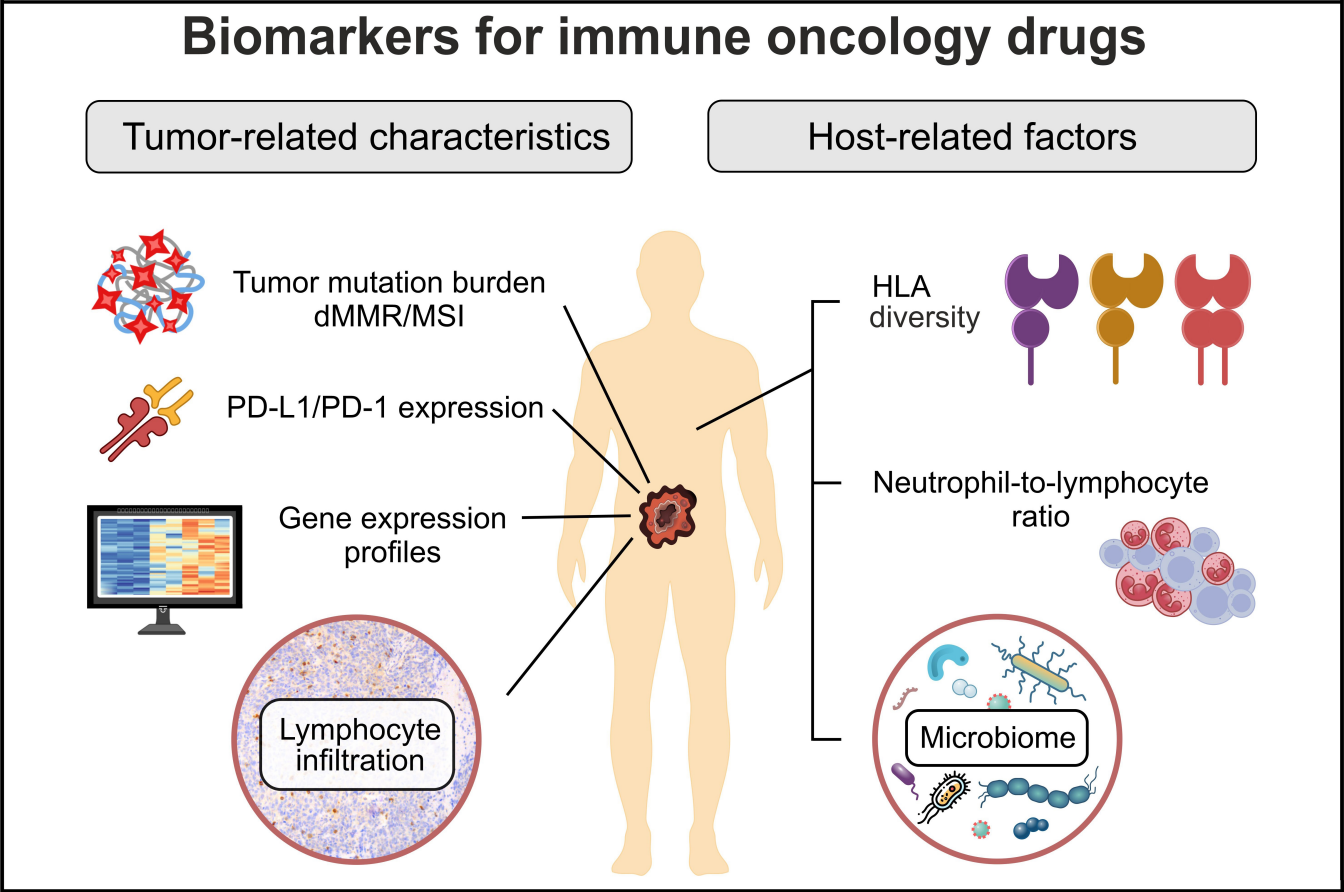
**Tumor- and host-related factors affecting the outcome of immune therapy.** HLA: human leucocyte antigens; dMMR: deficiency in mismatch DNA repair; MSI: microsatellite instability

Presently, all approved ICI therapies are targeted mainly towards PD-L1/PD-1 axis (Table 1). Anti-PD-1 or anti-PD-L1 antibodies constitute the backbone of the ICI treatment. In some instances, PD-L1/PD-1 inhibition is supplemented by targeting other immune regulators (CTLA-4 or LAG-3), or by the addition of some standard-of-care drugs (cytotoxic compounds, multikinase inhibitors, bevacizumab, etc.). The success of immune therapy presumably depends on the interplay between tumor-related factors and host-related factors (Figure 1). The former category of predictive markers includes various surrogates of tumor antigenicity as well as characteristics of the immune status of the tumor microenvironment. Host-related factors are more complex, being dependent on the general condition of the patient, the overall capacity of the immune system, and a number of confounding parameters, such as various comorbidities, microbiome composition, etc. For the time being, only the analysis of tumor parameters has already been incorporated into clinical practice. It is currently utilized for some although not all single-agent immune therapies, while the use of ICIs in combination with other drugs is usually not guided by biomarker testing (Table 1).

**Table 1:** Biomarker-tailored and biomarker-independent therapies for immune oncology drugs

**Tumor type**	**PD-1 inhibitors**			**PD-L1 inhibitors**		
	**Pembrolizumab**	**Nivolumab**	**Dostarlimab**	**Atezolizumab**	**Avelumab**	**Durvalumab**
Biomarker-tailored therapies for metastatic or unresectable disease						
NSCLC without ALK/EGFR alterations, 1st line	Single-agent, ≥ 1% PD-L1 positive tumor cells	In combination with ipilimumab, ≥ 1% PD-L1 positive tumor cells	-	Single-agent, ≥ 50% PD-L1 positive tumor cells, or PD-L1 positive immune cells covering ≥ 10% of the tumor area	-	-
NSCLC without ALK/EGFR alterations, previously treated	Single-agent, ≥ 1% PD-L1 positive tumor cells	-	-	-	-	-
HNSCC, 1st line	Single-agent, CPS ≥ 1	-	-	-	-	-
Triple-negative breast carcinoma, 1st line	In combination with chemotherapy, CPS ≥ 10	-	-	-	-	-
Esophageal carcinoma, previously treated	Single-agent, CPS ≥ 10	-	-	-	-	-
Gastric carcinoma, HER2-positive, 1st line	In combination with trastuzumab, platinum and fluoropyrimidines, CPS ≥ 1	-	-	-	-	-
Gastric carcinoma, previously treated	Single-agent, CPS ≥ 1	-	-	-	-	-
Urothelial carcinoma, cisplatin-ineligible	Single-agent, CPS ≥ 10	-	-	Single-agent, PD-L1 positive immune cells covering ≥ 5% of the tumor area	-	-
Cervical carcinoma, 1st line	In combination with chemotherapy, CPS ≥ 1	-	-	-	-	-
Colorectal carcinoma, 1st line	Single-agent, MSI-H/dMMR	-	-	-	-	-
Colorectal carcinoma, previously treated	Single-agent, MSI-H/dMMR	Single-agent or in combination with ipilimumab, MSI-H/dMMR	-	-	-	-
Endometrial carcinoma, 1st line	-	-	In combination with carboplatin and paclitaxel, following by single-agent, dMMR or MSI-H	-	-	In combination with carboplatin and paclitaxel, following by single-agent, dMMR
Endometrial carcinoma, previously treated	Single-agent, MSI-H/dMMR	-	Single-agent, dMMR	-	-	-
All tumor types (agnostic), previously treated	Single-agent, MSI-H/dMMR	-	Single-agent, dMMR	-	-	-
All tumor types (agnostic), previously treated	Single-agent, high TMB (≥ 10 mutations per megabase)	-	-	-	-	-
Biomarker-independent therapies for metastatic or unresectable disease						
Melanoma	Single-agent	Single-agent or in combination with ipilimumab or relatlimab	-	In combination with vemurafenib and cobimetinib for BRAF V600 mutated melanoma	-	-
NSCLC without ALK/EGFR alterations, 1st line	Non-squamous: in combination with pemetrexed and platinum; squamous: in combination with carboplatin and paclitaxel	In combination with ipilimumab and 2 cycles of platinum-doublet	-	In combination with chemotherapy and bevacizumab	-	In combination with tremelimumab-actl and platinum
NSCLC, previously treated	-	Single-agent	-	Single-agent	-	-
NSCLC, stage III, after chemo- and radiotherapy	-	-	-	-	-	Single-agent
SCLC, 1st line	-	-	-	In combination with carboplatin and etoposide	-	In combination with platinum and etoposide
SCLC, previously treated	Single-agent	-	-	-	-	-
Malignant pleural mesothelioma, 1st line	-	In combination with ipilimumab	-	-	-	-
HNSCC, 1st line	In combination with platinum and FU	-	-	-	-	-
HNSCC, previously treated	Single-agent	Single-agent	-	-	-	-
Esophageal carcinoma, 1st line	In combination with platinum and fluoropyrimidines	In combination with platinum and fluoropyrimidines, or in combination with ipilimumab	-	-	-	-
Esophageal squamous cell carcinoma, previously treated	-	Single-agent	-	-	-	-
Gastric carcinoma, 1st line	In combination with platinum and fluoropyrimidines	In combination with platinum and fluoropyrimidines	-	-	-	-
Biliary tract carcinoma	In combination with gemcitabine and cisplatin	-	-	-	-	In combination with gemcitabine and cisplatin
Urothelial carcinoma, 1st line	In combination with enfortumab vedotin	-	-	-	-	-
Urothelial carcinoma, platinum-ineligible	Single-agent	-	-	Single-agent	-	-
Urothelial carcinoma, previously treated	Single-agent	Single-agent	-	-	Single-agent	-
Classical Hodgkin lymphoma, previously treated	Single-agent	Single-agent	-	-	-	-
Primary mediastinal large B-cell lymphoma	Single-agent	-	-	-	-	-
Hepatocellular carcinoma, 1st line	-	-	-	In combination with bevacizumab	-	In combination with tremelimumab-actl
Hepatocellular carcinoma, previously treated	Single-agent	In combination with ipilimumab	-	-	-	-
Merkel cell carcinoma	Single-agent	-	-	-	Single-agent	-
Renal cell carcinoma, 1st line	In combination with axitinib or lenvatinib	In combination with ipilimumab or cabozantinib	-	-	In combination with axitinib	-
Renal cell carcinoma, previously treated	-	Single-agent	-	-	-	-
Endometrial carcinoma, without MSI-H/dMMR, previously treated	In combination with lenvatinib	-	-	-	-	-
Cutaneous squamous cell carcinoma	Single-agent	-	-	-	-	-
Alveolar soft part sarcoma	-	-	-	Single-agent	-	-
Neoadjuvant therapy						
NSCLC	In combination with platinum containing chemotherapy	In combination with platinum doublet	-	-	-	-
Triple-negative breast cancer	In combination with chemotherapy	-	-	-	-	-
Adjuvant therapy						
Melanoma	Single-agent	Single-agent	-	-	-	-
Urothelial carcinoma	-	Single-agent	-	-	-	-
Esophageal carcinoma	-	Single-agent	-	-	-	-
NSCLC	Single-agent	-	-	Single-agent, ≥ 1% PD-L1 positive tumor cells	-	-
Renal cell carcinoma	Single-agent	-	-	-	-	-

CPS: combined positive score; dMMR: deficiency in mismatch DNA repair; HNSCC: head and neck squamous cell carcinoma; MSI-H: high-level microsatellite instability; NSCLC: non-small-cell lung cancer; SCLC: small-cell lung cancer; TMB: tumor mutation burden; FU: fluorouracil

## Tumor-related characteristics

### PD-L1 expression

A predictive role of PD-L1 expression was initially shown in a nivolumab clinical study, in which 9/25 (36%) PD-L1 positive but none of 17 PD-L1 negative tumors demonstrated objective response. PD-L1 status was assessed by an immunohistochemical (IHC) analysis of tumor cells, with 5% of stained cells taken as a cut-off [3]. Subsequent investigations complicated the field. Although PD-L1 is a major ligand interacting with the PD-1 receptor, there are other molecules involved in the modulation of PD-1 activity. Some studies suggested that the expression of PD-1 rather than PD-L1 is a marker of efficacy of anti-PD-1 therapeutic antibodies [4]. Furthermore, PD-L1 expression appears to be predictive only for selected cancer types, therefore this biomarker does not have an “agnostic” significance [5].

There are technical nuances related to the determination of PD-L1 status. The source of this ligand is not confined to cancer cells, as tumor microenvironment, particularly immune cells (ICs), may produce a significant amount of PD-L1 and thereby render peritumoral immune suppression. Consequently, several clinical studies considered PD-L1 status both for tumor cells and for their microenvironment. PD-L1 analysis of tumor cells was similar across various investigations and relied on the calculation of the proportion of stained tumor cells. Several atezolizumab trials utilized an additional parameter, so-called IC score defined as an area occupied by PD-L1 expressing ICs in relation to the total tumor area. In addition, some pembrolizumab studies relied on the combined positive score (CPS), which was obtained by dividing the number of PD-L1 positive tumor cells, lymphocytes, and macrophages by the total number of tumor cells [6]. There was no clearly articulated mechanistic rationale why some data sets relied only on the status of tumor cells, while others considered ICs as well. Furthermore, all thresholds between “positive” and “negative” cases were obtained using post hoc analysis (Table 1).

Several PD-L1 IHC assays have been proposed for clinical use, each coupled to a particular PD-1 or PD-L1 inhibitor. It is assumed that Dako 22C3, Dako 28-8, and Ventana SP263 tests produce essentially similar results, while the Ventana SP142 assay utilized for atezolizumab studies consistently reveals a lower percentage of both tumor cells and ICs. While inter-assay and interobserver reproducibility is acceptable for PD-L1 analysis of tumor cells, it is insufficient for ICs [6–9]. Many nuances in laboratory procedures are critical for the consistency of PD-L1 testing, therefore, the real-world picture may substantially differ from the results obtained in well-controlled investigations [10–12]. It is fair to acknowledge that the interlaboratory and interobserver reproducibility of PD-L1 analysis appears to have improved over time, as recent investigations produced more encouraging results than earlier comparative studies [13, 14]. After all, only a minority of indications for immune oncology drugs rely on PD-L1 testing, while in many instances anti-PD-L1/PD-1 containing therapies are administered irrespective of PD-L1 status (Table 1).

There is room to improve the laboratory techniques for PD-L1 detection. It is difficult to explain why even anti-PD-L1 targeted drugs demonstrate a substantial rate of responses in seemingly PD-L1 negative tumors [15, 16]. PD-L1 glycosylation may compromise its interaction with diagnostic antibodies, and chemical removal of glycans results in the improvement of sensitivity of PD-L1 IHC analysis [17]. These data are supported by reports, which demonstrate that many tumors express significant levels of PD-L1 mRNA in the absence of IHC-detectable protein staining [18]. The predictive value of PD-L1 mRNA expression has been confirmed in several transcriptomic studies [19–21]. Interestingly, fluorescence-based detection of PD-L1/PD-1 complexes appears to outperform PD-L1 testing alone [22].

### Microsatellite instability

Some tumors are characterized by the increased number of somatic mutations due to failure of DNA repair. The most known example is high-level microsatellite instability (MSI-H), i.e., accumulation of small deletions and insertions in short nucleotide repeats due to deficiency in mismatch DNA repair (dMMR). MSI-H is a historical definition, which emerged in 1990s when researchers attempted to discriminate between tumors with high and moderate numbers of alterations in microsatellites; nowadays, the terms MSI and MSI-H are used interchangeably. Most microsatellite sequences are non-coding, therefore, changes in their length do not have an apparent contribution to tumor pathogenesis but serve merely as a diagnostic marker for dMMR. However, some microsatellites are located within exons; furthermore, the impact of dMMR is not limited only to changes in microsatellite length but extends to other types of genetic alterations. Hence, dMMR/MSI-H tumors are characterized by dramatic elevation of the number of coding mutations. This renders increased antigenicity of tumor cells, consequently, dMMR/MSI-H carcinomas have lower relapse rates after radical surgery and are highly sensitive to immune oncology drugs. dMMR/MSI-H initially gained acceptance for the treatment of metastatic colorectal cancer and then received a status of “agnostic” marker (Table 1) [23]. In addition, there are several highly successful neoadjuvant and adjuvant ICI trials utilizing dMMR/MSI-H tumors [23–26].

dMMR/MSI-H is apparently the most straightforward biomarker for ICI therapy. For example, preoperative administration of nivolumab and relatlimab produced major pathologic responses in 92% of dMMR colorectal cancer patients [24]. However, dMMR/MSI-ICI matching is relevant only to a small subset of tumors. dMMR/MSI-H is characteristic approximately for 5–10% of colorectal, gastric and biliary tract carcinomas as well as for 15–20% of endometrial malignancies, while its incidence in most other tumor types is below 1%. For example, MSI-H almost never occurs in lung tumors, breast carcinomas, melanomas, etc. [27–30]. Although dMMR/MSI-H is commonly promoted as “agnostic” indication for ICI, the feasibility of its routine evaluation in other than gastrointestinal or endometrial tumors is questionable. Furthermore, some tumor types demonstrate relatively low efficacy of ICI therapy despite the presence of dMMR/MSI-H [31].

The techniques for MSI-H determination were developed three decades ago and, from the laboratory perspective, the discrimination between MSI-H and non-MSI-H carcinomas is not complicated. Nevertheless, misclassification of tumors with regard to MSI-H status is not infrequent in clinical practice [32]. The most established approach for MSI-H testing relies on the detection of length changes in 5 quasi-monomorphic mononucleotide repeats (BAT25, BAT26, NR21, NR24, and NR27). This technique requires equipment for capillary electrophoresis and basic skills in molecular biology. An alternative approach, which is compatible with a standard morphological laboratory, is based on immunohistochemical detection of relevant mismatch DNA repair proteins (MLH1, MSH2, MSH6, and PMS2). The dMMR status is assigned to tumors demonstrating either paired depletion of MLH1/PMS2 or MSH2/MSH6, or isolated loss of MSH6 or PMS2. Historically, these approaches were developed for the analysis of colorectal cancer as well as for the selection of patients with suspicion of Lynch syndrome. Colorectal cancer data sets demonstrate generally good concordance between PCR-based MSI-H testing and IHC analysis for dMMR, so these methods appear to be interchangeable. However, studies on Lynch syndrome revealed that some non-gastrointestinal dMMR tumors, which presumably have a low rate of cell proliferation, do not have widespread microsatellite instability and, therefore, are unlikely to be highly antigenic [33–35]. The majority of next-generation sequencing (NGS) services now include MSI-H status in their reports. However, almost all currently utilized NGS panels were purposely designed for the analysis of exonic regions of actionable genes and, therefore, patterns of mutations they reveal are enriched for potentially functional events; thorough testing for non-coding microsatellite markers may provide a more unbiased snapshot of the status of nucleotide repeats [34, 36]. Technical nuances of dMMR/MSI-H determination deserve to be closely monitored in ICI studies on non-colorectal cancer types.

### Tumor mutation burden

Tumor mutation burden (TMB) was initially defined as the total number of non-synonymous somatic mutations present in the genome of transformed cells [37]. Subsequent studies revealed that small insertions and deletions are generally more antigenic than amino acid substitutions. Accumulation of indels is particularly characteristic of renal cell and bladder carcinomas, which are well known for their responsiveness to immune therapy [38, 39]. Since whole exome sequencing (WES) is not always feasible in daily clinical practice, many NGS diagnostic services offer more accessible tests in which the approximate TMB value is calculated based on the analysis of a few hundred genes. For example, the agnostic approval of pembrolizumab relied on the TMB value ≥ 10 mutations per megabase estimated by the FoundationOne CDx test [40]. The mechanistic basis underpinning the predictive value of TMB assumes a general correlation between the increased number of somatic mutations and the high amount of antigens, hence high TMB tumors are more likely to be immunogenic than low-TMB neoplasms. There are two causes for increased TMB. First, high TMB is characteristic of carcinogen-induced tumors, for example, smoking-related lung carcinomas or melanomas caused by excessive exposure to ultraviolet. Secondly, alterations in DNA replication machinery or repair may result in the accumulation of somatic mutations [34]. In this respect, it is important that the proof-of-concept ARETHUSA clinical trial demonstrated that cytotoxic treatment with temozolomide may modify DNA repair and TMB in otherwise ICI-resistant carcinomas, and eventually prime these tumors to immune therapy [41].

Undoubtedly, tumors with significantly elevated TMB are likely to respond to ICI therapy. Surrogates for increased TMB may diminish the need for laboratory TMB testing. For example, history of regular smoking correlates both with high TMB and with lung cancer sensitivity to ICI, therefore, it may reliably guide treatment decisions [37, 42]. Similarly, the location of melanoma on skin areas affected by sunburns suggests both an excessive number of mutations and a high probability of benefit from immune therapy [43]. MSI-H is an excellent indicator of ultra-high TMB, however, MSI-H testing is significantly more rapid and accessible compared with TMB determination [34, 44]. Some tumors, particularly colorectal and endometrial carcinomas, carry hot-spot alterations in *POLE* gene encoding for DNA polymerase; *POLE*-mutated malignancies usually have high TMB and are responsive to ICI [45, 46]. Similar relationships are observed for malignancies with inactivation of *MUTYH* gene, which encodes for base excision repair enzyme [47, 48].

It is essential to recognize that the ICI responders from most TMB-tailored trials were significantly enriched by the categories of cancer patients described above. The clinical value of TMB can be questionable if tumors with an overt history of carcinogen exposure and malignancies with MSI-H or *POLE* mutations are excluded. For example, the efficacy of pembrolizumab-containing therapy correlated with high TMB in a gastric cancer study; however, 44% of high TMB patients were MSI-positive, and the exclusion of these subjects from the analysis resulted in the attenuation of the observed correlations [49]. Some tumor types do not demonstrate an association between increased TMB and ICI responsiveness, as exemplified by the data obtained on breast carcinomas or brain neoplasms [50–52]. Perhaps, some controversy can be resolved by increasing the threshold for high TMB [53]. Importantly, systematic studies on TMB distribution revealed a significant number of tumors, which have “ultrahigh” TMB in the absence of identified causative factors [44]. These outliers, which are observed across virtually all cancer types, deserve comprehensive investigation regarding their frequencies in different categories of patients, clinical features, underlying genetic mechanisms and sensitivity to ICIs.

Not all non-synonymous mutations generate neoantigens. For example, some mutations result in nonsense-mediated RNA decay or decreased stability of the corresponding protein. Transcriptome sequencing may be more relevant than DNA analysis for the evaluation of potential tumor immunogenicity [54, 55]. Furthermore, the antigenicity of mutated peptides depends not only on the character of amino acid sequence changes, but also on the ability of individual human leucocyte antigens (HLA) molecules to recognize these mutations and present them to the immune system. Matching of WES data to personal HLA genotypes can be achieved using several software tools [55–57].

### Mutations in selected genes

Some studies demonstrated associations between mutations in particular genes and ICI efficacy. The best-known example is *KRAS* G12C substitution in lung carcinomas [58]. *KRAS* G12C mutation, but not other common types of *KRAS* alterations, demonstrates a tight association with the history of smoking [59]. Consequently, *KRAS* G12C merely serves as an indicator of the tobacco-related nature of lung malignancy and high TMB; so, in some instances, it can be considered together with or instead of self-reported smoking history [42, 60]. Importantly, *KRAS* G12C amino acid change occurs at lower frequencies in other cancer types, but it does not have “agnostic” correlations with tumor immunogenicity or ICI responsiveness [61, 62]. Earlier clinical trials revealed associations of somatic mutations in *SERPINB3* or *SERPINB4* genes with both high TMB and improved efficacy of CTLA-4 inhibition in melanoma [63]. However, these results have not been subjected to rigorous replication studies. Some data suggest the role of mutations in *KEAP1*, *LKB1* (*STK11*), *ARID1A*, *PTEN*, and several other genes, but the translational relevance of these findings is unclear [58, 64–66]. There is a critical mass of data suggesting that Epstein-Barr virus (EBV) associated cancers are responsive to ICI, therefore, EBV testing deserves to be considered in some tumor types [67, 68].

### Lymphocyte infiltration

Tumors demonstrate significant diversity with regard to interaction with ICs. Some cancers are characterized by increased lymphocyte infiltration (“immune-hot”), so their treatment may require only functional activation of these cells. Other malignancies appear to have expelled ICs, so they are located on tumor margins (“immune-excluded”); these lymphocytes may fight neoplastic growth when permitted to enter the tumor milieu. “Immune-cold” neoplasms, which do not have ICs either in the tumor core or in the periphery, are probably not good candidates for ICI therapy.

Several studies suggest that increased tumor infiltration by ICs is a favorable predictor for ICI therapy [69–71]. However, this is an oversimplification because tumor stroma may contain both “good” lymphocytes which are ready to fight against tumor cells and “bad” ICs which either do not have antitumor potential or even render negative regulation of immune response. Additionally, not a mere content of lymphocytes but a proper equilibrium between various IC types infiltrating the tumor appears to be essential for ICI efficacy. Current studies employ tools for discriminating between “good” and “bad” tumor-infiltrating cells and use sophisticated scoring approaches to account for spatial organization of cancer lumps [72–76]. Immunoscore is the best-known assay of this type: it evaluates the content of CD-3-positive and CD-8-positive T-cells in the tumor core and invasive margin by analyzing digital images with specially designed software [75, 76]. The complexity of the analysis of tumor microenvironment complicates its translation into clinical practice.

### Gene expression profiles

There is a multitude of genes with known roles in the regulation of immune response. Both hypothesis-driven and transcriptomic studies revealed various gene expression profiles (“signatures”) associated with ICI efficacy [77–81]. A major limitation of these studies is the lack of practical avenues for their replication: indeed, almost all published scores or indices are intended mainly for in-house use, be it research activities or commercial diagnostic services. ICI-related expression profiles reflect mainly the functional status of the immune tumor microenvironment, particularly T-cell activation and interferon-gamma signaling [73, 78, 82]. Consequently, the results of these studies are potentially influenced by the spatial organization of the tumor, as different parts of the latter are likely to produce different snapshots of gene transcripts. For example, a renal cell carcinoma study revealed distinct transcriptomic profiles in tumor areas with positive and negative PD-L1 status of T-cells [83]. Despite all these caveats, gene expression profiles are viewed as a viable substitute for the scoring of ICs, as they can be subjected to some degree of automatization and less prone to interobserver variability [2, 84].

## Host-related factors

### Microbiome

The ICI-predictive role of the microbial composition of the gut was initially demonstrated in mouse experiments. Sivan et al. [85] observed differences in antitumor immunity in genetically identical mice with distinct intestinal microbiomes. They revealed that *Bifidobacterium* species played a significant role in immune response regulation; importantly, the efficacy of anti-PD-L1 therapy was dramatically improved by its supplementation with *Bifidobacterium* preparations. Vétizou et al. [86] suggested the role of *Bacteroides* in determining the tumor response to anti-CTLA4 blockade. Subsequently, several studies in cancer patients revealed that antibiotic therapy compromises the efficacy of ICIs and the pattern of intestinal microbes may differ between ICI responders and non-responders [87, 88]. Some data sets emphasized the role of particular microbal species, for example, *Bifidobacterium longum*, *Enterococcus faecium*, *Bifidobacterium pseudocatenulatum*, *Collinsella aerofaciens*, *Akkermansia muciniphila*, *Bacteroides*, *Ruminococcaceae*, *Agathobacter*, *Prevotella*, *Lachnospiraceae*, *Roseburia*, butyrate-producing bacteria, etc. [87–95]. In addition, there are data suggesting that not only particular species but also the diversity of microorganisms as well as some equilibrium in their abundance are essential for the success of ICI therapy [89, 91, 92, 96]. Strikingly, clinical trials demonstrated that fecal microbiome transplantation from ICI responders to non-responders may restore tumor sensitivity to ICI [97, 98].

The mechanisms underlying the influence of commensal microbes on ICI immunity are currently under investigation. Cross-reactivity between bacterial and tumor antigens may support “education” of ICs. The healthy composition of microbiome is essential for the baseline proficiency of host defense mechanisms. Bacteria populating the human body produce a number of metabolites, some of which are essential for antitumor immunity [99, 100].

Despite the overall promise of microbiome research, there are no relevant laboratory assays allowing the prediction of response or resistance in a given individual. Insufficient interstudy agreement may be attributed to several confounding parameters, such as geographic and dietary variations, technical nuances in sample collection and NGS analysis, differences in analyzed tumor types and treatments, etc. [95]. Despite these limitations, several interventional trials attempted the use of microbiome-modifying approaches to improve the efficacy of ICI therapy. Dizman et al. [101] utilized *Clostridium butyricum* preparation in a randomized phase 1 study involving patients with metastatic renal cell carcinoma; this supplementation was accompanied by evident improvement of the progression-free survival (12.7 vs. 2.5 months, *p* = 0.001). In a similarly designed study, subjects receiving *Bifidobacterium* live bacterial product in addition to cabozantinib and nivolumab therapy experienced a higher rate of responses as compared to controls [14/19 (74%) vs. 2/10 (20%), *p* = 0.01] [102]. However, some microbiome-related studies call for caution. For example, Glitza et al. [103] supplemented immune therapy with a bacterial formulation in a phase 1b melanoma randomized trial and observed numerically higher response rates (4/6, 67%) in patients receiving placebo versus subjects taking probiotics (2/8, 25%). Spencer et al. [104] (2021) revealed that self-reported use of probiotics certainly did not improve but even tended to compromise the outcomes of ICI therapy in melanoma patients.

### Neutrophil-to-lymphocyte ratio

Neutrophil-to-lymphocyte ratio (NLR) was suggested to be of clinical significance in 2001 by Dr. Roman Zahorec [105]. This index is an easily accessible parameter, and many ICI studies revealed that patients with high NLR are poor responders to immune therapy [106–112]. It is assumed that NLR reflects the balance between pro-tumoral inflammation and anti-tumoral immune defense [113].

Inflammation and antitumor immunity are closely related processes, which have significant overlap in the involved molecules and cell subsets. Acute inflammation plays an essential role in the immediate response to infections and promotes the subsequent emergence of adaptive immunity. However, in many instances host defense mechanisms fail to eliminate foreign antigens, so the inflammation becomes chronic. Continuing inflammation appears to induce some degree of immune tolerance thus preventing autoimmune reactions. Mature tumors are by definition associated with chronic antigen exposure. Furthermore, cancer patients often have significant comorbidities and age-related inflammatory derangements. These factors may result in down-regulation of antitumor immunity [114].

Several data sets suggest that consideration of NLR together with other ICI predictive factors may provide a meaningful tool for identifying patients with a particularly high or particularly low probability of benefit from immune therapy [108–111]. It should be noted that although available studies provide a relatively consistent picture, the threshold between favorable and unfavorable NLR was a subject of substantial variations. More importantly, it is unclear whether cancer patients with high baseline NLR may benefit from some supportive therapy aimed at normalizing homeostasis.

### HLA

HLA genes encode for peptides, which recognize foreign proteins and present their antigenic fragments to T-cells. HLA includes class I and II genes, with the former playing a primary role in the detection of cancer cells. The function of HLA class I peptides is to bind endogenous antigens produced by mutated proteins and to transport them to the cell surface for display to the immune system [11, 115].

HLA class I genomic region consists of three loci (A, B, and C). Each of the above genes is highly polymorphic. Human studies usually demonstrate 2–3 dozen relatively frequent A and C alleles in every population analyzed, and this number is approximately twice as high for the B locus. Importantly, HLA alleles differ from each other by the spectrum of recognizable foreign amino acid motifs. Consequently, a given mutated protein may be potentially detectable by one HLA genotype, but remain invisible to immunity in an individual with another HLA composition [116]. This variability explains well-known associations between HLA allelism and predisposition to various immune-related diseases, including infections, autoimmune disorders, cancer, etc. [115, 117–119]. The majority of people have distinct alleles for A, B, and C genes each, and these subjects are likely to have an advantage with regard to the spectrum of recognizable antigens. Approximately, one out of ten individuals are homozygous for A or C loci, and approximately one out of twenty subjects have identical maternal and paternal alleles for B locus. These estimates are even higher when closely related gene variants are united in HLA supertypes [118].

There are data sets demonstrating that the reduced HLA class I diversity, i.e., homozygosity in *HLA-A*, *HLA-B*, or *HLA-C* genes, is a negative predictive factor for response to immune therapy, as the best responses are observed in individuals carrying distinct alleles in all of the above genes [120–123]. However, the data are not consistent across studies [124, 125]. Comprehensive HLA genotyping remains complicated even with the invention of NGS, as it requires specific laboratory assays and software tools as well as profound expertise in HLA genetics. In addition, somatic status of HLA molecules should be investigated, as some tumors adapt to immune therapy by the loss of HLA expression, so the mutated proteins become hidden from the host defense [114, 115].

## Conclusions

Highly elevated number of somatic mutations, and consequently, cancer-specific antigens, is apparently the most convincing indicator of potential tumor sensitivity to immune therapy. However, this feature is relatively rare. The PD-L1 biomarker has significant limitations, therefore, the attempts to supplement this parameter by the analysis of gene expression profiles and IC infiltration may improve patients’ selection for immune oncology drugs. While tumor-related parameters have already been extensively studied, comprehensive analysis of host-related factors is not always included in relevant clinical trials. Perhaps, consideration of one or a few parameters is not sufficient for reliable prediction of the efficacy of immune therapy. There is a need for tools, which will be able to account for the multitude of tumor and host characteristics, such as mutation profile, transcriptome data, microbiome composition, HLA genotype, various phenotypic characteristics of the immune system, pattern of comorbidities, concurrent treatments, etc., and to provide integrative analysis of this complexity. Artificial intelligence (AI) tools may significantly facilitate the interpretation of multiple data sets and develop new approaches for the personalization of immune therapy. Categorization of patients for potential responders and non-responders to ICI is not the only outcome of these efforts. Research on biomarkers for cancer immune therapy offers the hope of discovering modifiable tumor and patient characteristics, which can be utilized for educating the immunity against transformed cells.

## Abbreviations

dMMR: deficiency in mismatch DNA repair
EBV: Epstein-Barr virus
HLA: human leucocyte antigens
ICIs: immune checkpoint inhibitors
ICs: immune cells
IHC: immunohistochemical
MSI-H: high-level microsatellite instability
NGS: next-generation sequencing
NLR: neutrophil-to-lymphocyte ratio
TMB: tumor mutation burden
WES: whole exome sequencing

## Declarations

### Copyright

© The Author(s) 2025.
